# The efficiency of health resource allocation and its influencing factors: evidence from the super efficiency slack based model-Tobit model

**DOI:** 10.1093/inthealth/ihac054

**Published:** 2022-08-13

**Authors:** Jing Gong, Leiyu Shi, Xiaohan Wang, Gang Sun

**Affiliations:** Department of Hospital Management, Tsing Hua University, Shenzhen Campus, Shenzhen 518000, China; Department of Health Policy and Management, Bloomberg School of Public Health, Johns Hopkins University, Baltimore, MD 21205, USA; Department of Health Management, School of Health Management, Southern Medical University, Guangzhou, Guangdong 510515, China; Department of Health Management, School of Health Management, Southern Medical University, Guangzhou, Guangdong 510515, China

**Keywords:** healthcare resource, Malmquist index model, productivity, super efficiency SBM model, Tobit model

## Abstract

**Background:**

This study aims to analyze the health resource allocation efficiency in Sichuan Province from 2010 to 2018 and provide other countries with China's experience.

**Methods:**

We used the super efficiency slack based model (SBM) model and Malmquist index to analyze the super efficiency and inter-period efficiency of health resource allocation in 19 cities in Sichuan Province from 2010 to 2018 and propose the input-output optimization scheme of health resource allocation in 2018. Finally, the Tobit model was used to estimate the influencing factors of health resource allocation efficiency.

**Results:**

The total allocation of health resources in Sichuan Province was increasing in addition to the total number of visits from 2010 to 2018. The super efficiency SBM results identified that the sample's average score was between 0.651 and 3.244, with an average of 1.041, of which 15 cities had not reached data envelopment analysis effectiveness. According to the Malmquist index, the average total factor productivity index of Sichuan Province was 0.930, which showed an imbalance in resource input, and its fluctuation was mainly related to the technological progress index and scale efficiency. The efficiency score was affected by the average annual income of residents, population density and education level.

**Conclusions:**

The amount of health resource allocation in Sichuan Province had shown an overall upward trend since 2010. However, resource allocation efficiency was not high, and there were problems such as significant regional differences, insufficient technological innovation capabilities and unscientific allocation of resource scale. To optimize the resource allocation structure, we suggest that the relevant departments pay attention to the impact of natural disasters, the average annual income of residents, population density and education level on efficiency to allocate health resources scientifically.

## Introduction

Health resources are the avenue for residents to obtain health services, and their allocation efficiency is related to people's health and the development of health services.^1^ At present, there is a problem of low efficiency in resource management in most developing countries,^2^ and how to rationally allocate health resources has gradually become a common topic in the world.^3^ To optimize the allocation of resources, China started a new medical reform in 2009 and achieved certain results. The proportion of residents with basic medical insurance increased from <10% to 95%, and China basically achieved full medical insurance coverage.^4^ Since the new medical reform, the number of health resource inputs in China has shown an overall upward trend. From 2010 to 2018, the number of health institutions, beds and health technicians in China increased by 6.46%,75.57% and 49.87%, respectively.

Nevertheless, some scholars analyzed the resource allocation efficiency of 31 provinces and cities in China from 2010 to 2017 and found that only 10 provinces and cities achieved data envelopment analysis (DEA) effectiveness, with enormous regional differences.^5^ Consequently, although the number of health resource allocations in China is constantly increasing, its resource allocation efficiency is not high. There is still a long way from the full realization of everyone's goal of enjoying essential medical and health services.

People's demand for health services has been increasing, but resources are limited. For health decision-makers, the key to management is to determine whether the invested resources are used scientifically, and DEA is a better way to study resource allocation efficiency. The DEA method was introduced into the medical field in the 1980s.^6^ Because DEA can consider the multi-input and multi-output problems of multiple decision units in different periods and does not need to assume the production function, it has been widely used in the public health field, hospitals and primary healthcare centers.^7^

Although there have been plenty of articles in the past using the DEA model to analyze health resource allocation efficiency, these have some limitations. First, most authors used the radial DEA model, which might lead to errors in the research results due to random errors and environmental factors.^5^ Second, they only analyzed the efficient allocation of health resources and did not analyze the influencing factors. Third, these articles were not scientific in selecting indicators, such as selecting health institutions as input indicators and ignoring the homogeneity between them.^8^ The mixed use of absolute numbers and relative numbers was also common. Most of the literature uses bed turnover rate and the number of visits as input indicators simultaneously, which is unreasonable.^9^ Consequently, we made improvements on the basis of these articles.

China has long faced the problem of uneven regional development. In 2016, to further explore new ideas in line with China's medical reform, Sichuan Province was established as a comprehensive medical reform pilot province. Sichuan Province is located in southwestern China, with a permanent population of 83.41 million, an area of 486 052 square kilometers and a regional gross domestic product (GDP) of 4.067813 billion yuan in 2018. Sichuan Province has been committed to improving the accessibility and fairness of health resources for residents. It has been 5 y since Sichuan Province implemented comprehensive medical reform. This study analyzed the efficiency of health resource allocation in Sichuan Province to establish the reform's effectiveness. The primary purpose of the study is to: (1) assess the efficiency of health resource allocation in Sichuan Province during the 2010–2018 medical reform period; (2) based on input-output slack variables, propose an optimization plan for resource allocation in 2018; (3) research the influencing factors of technical efficiency; and (4) provide for other countries China's experience in the optimal allocation of resources.

## Methods

### DEA

DEA is a non-parametric method to measure the relative efficiency of homogeneous decision-making units (DMUs) with multiple input and output variables,^10^ which is divided into the radial model, non-radial model and extended model.^11^ The radial model assumes that the input and output change proportionally, and it mainly includes two types: Charnes, Cooper, and Rhodes (CCR) and Banker, Charnes, and Cooper (BCC). The non-radial model mostly uses the slack based model (SBM) model.^12^ The extension model is represented by the super efficiency DEA model. The maximum super efficiency score is no longer limited to 1, which solves the problem that the radial model cannot compare effective DMUs.^13^ The super efficiency SBM (SE-SBM) model proposed an input-output optimization scheme based on slack variables, which has been widely used in recent years.

Regardless of whether it is an input- or output-oriented perspective, the model's slack will be biased, so this study chooses non-angle oriented. Due to the uncontrollability of output, this study uses the SE-SBM model to analyze Sichuan's health resources from 2010 to 2018 based on the variable return to scale assumption. The basic ideas^14^ are as follows:

$$\begin{eqnarray*}\rho \ = { min} \frac{{\frac{1}{m}\mathop \sum \nolimits_{a = 1}^m \frac{{S_a^ - }}{{{x}_{ak}}}}}{{\frac{1}{s}\mathop \sum \nolimits_{c = 1}^s \frac{{S_c^ + }}{{{y}_{ck}}}}}\end{eqnarray*}$$

$$\begin{eqnarray*}st.\displaystyle\mathop \sum^{n}_{\begin{array}{@{}*{1}{c}@{}} {b = 1}\\{b \ne k} \end{array}} {x}_{ab}{\lambda }_b - S_a{}^ {-} \le {x}_{ak}\end{eqnarray*}$$

$$\begin{eqnarray*}st.\displaystyle\mathop \sum^n_{\begin{array}{@{}*{1}{c}@{}} {b = 1}\\ {b \ne k} \end{array}} {y}_{cb}{\lambda }_b + S_c^ + \ge {y}_{ck}\end{eqnarray*}$$

$$\begin{equation*}\quad\quad\quad\quad\quad\quad\,\,\,\,\,\,\,\,{\lambda }_b,S_a^ - ,S_c^ + \ge 0,\forall a,\!c,\!b.
\end{equation*}$$

ρ represents the super efficiency value, where >1 indicates that the DMU is valid and <1 indicates that the DMU is invalid. x_κ_ and y_κ_ represent the input and output values of DMU, while x_ab_ and y_cb_ represent the a-th input and c-th output of the b-th DMU, respectively. λ is the weight value, and $S_a^ - $ and $S_c^ + $ represent the slack variables of input and output, respectively.^14^

### Malmquist index

Because the organization may perform well in a certain period and not well in another period, it is necessary to compare the organization's efficiency in different periods.^15^ Because the radial model can only compare the same period's efficiency, the Malmquist index is needed to compare inter-period efficiency. The Malmquist index was proposed in 1953^16^; Fare et al.^17^ combined it with the radial DEA model to propose a Malmquist index method for cross-period evaluation of production efficiency. It fills the gap that the radial DEA model cannot dynamically analyze efficiency.^18^ Its basic ideas are as follows:

$$\begin{equation*}
TFPCH\ = \ EFFCH*TECHCH
\end{equation*}$$

$$\begin{equation*}
EFFCH\ = \ PECH*SECH.
\end{equation*}$$

TFPCH reflects the change of total factor productivity (TFP) between t and t+1. TFPCH>1 means productivity increases; TFPCH<1 means productivity decreases.

### Tobit regression

Because the super efficiency value belongs to the restricted dependent variable and is divided into different stages, the Tobit regression model is used to avoid the deviation caused by the least square method regression model.^19^ Tobit regression was proposed in 1958, using the maximum likelihood method as the guiding ideology to randomly select n groups of samples to estimate its maximum probability.^20^ Based on the SE-SBM model, we used the super efficiency value as the dependent variable and used the influencing factors as the independent variable. Tobit models are:

$$\begin{equation*}Yi\ = \ \beta iXi + \varepsilon i,\end{equation*}$$

where $Yi{}^* $ is the dependent variable, $Xi\ $is the explanatory variable, $\beta i $ is the coefficient of explanatory variable and $\varepsilon i $ obeys (0, *σ*) distribution, $i $ = 1, 2…n.

### Data and variable

#### Data source

The data come from the 2010–2018 *Statistical Yearbook of Sichuan Province*^21^ and *Health Yearbook of Sichuan Province*.^22^ This article is a descriptive retrospective study to investigate the resource allocation of Sichuan Province from 2010 to 2018. Among them, the data of Ganzi Tibetan Autonomous Prefecture and Liangshan Yi Autonomous Prefecture are partially missing, so the remaining 19 cities in Sichuan Province were selected. Each city serves as a DMU.

#### Variable selection

The indicators are selected based on the literature reviewed above and the credibility of the results (the sum of input and output indicators should be less than or equal to the value of the total DMU).^5,8,9,11,23^ Although the bed turnover rate is used frequently in China, some articles pointed out that absolute numbers and relative numbers should not be mixed,^9^ so the current study did not include this indicator. Furthermore, because DEA is a method of efficiency, this study's output indicators do not include quality indicators such as mortality and cure rate.^24^

For input indicators we selected health technicians, total annual expenditure and the actual number of beds to represent information on three aspects: personnel, funding and facilities. For output indicators, we selected the total number of visits and discharges to reflect outpatient and inpatient services.

The influencing factors are selected according to the four aspects of economy, population, education and urbanization level.^5,25^ First, economic factors are reflected by per capita GDP and the average annual income of residents. Second, the population factor is reflected by population density. Third, the level of education is defined as the number of graduates from regular high schools. Fourth, the level of urbanization is defined as the proportion of the urban population in the total population. The dependent variable is the efficiency value obtained by super efficiency SBM. Table 1 shows the definitions of the selected variables.

**Table 1.** Definitions of the selected variables^21,22^

Type	Variable	Definition	Unit
Input	Number of health technicians	Health technicians include licensed physicians, licensed assistant physicians, registered nurses, pharmacists, laboratory technicians, radiologists, health supervisors and interns.	person
	Number of beds	The number of fixed beds (non-compiled) at the end of the year.	quantity
	Total expenditure	The cost and loss of funds incurred by the unit in carrying out business and other activities.	yuan
Output	Total number of visits	The total number of people in all diagnosis and treatment work.	person
	Number of discharges	The number of people discharged from hospital after hospitalization.	person
Influencing factor	PGDP	Per Capita Gross Domestic Product	yuan
	The average annual income of residents	The average per capita wage during a certain period of time for employed persons.	yuan
	Urbanization rate	The proportion of the permanent population of a country to the total population of the country.	%
	Population density	The number of people per square kilometer.	person/sq.km
	Education	Graduates of Regular Senior Secondary high schools	person

#### Data analysis

Excel (Redmond, WA USA) was used to enter the data, SPSS24.0 (IBM, USA) to calculate the descriptive statistics, DEA-SOLVER Pro5.0 to calculate the super efficiency value, DEAP 2.1 (Queensland, Australia) to calculate the Malmquist index and Stata16.0 (Stata Corporation, College Station, TX, USA) to analyze the influencing factors.

## Results

### Descriptive analysis

Table 2 shows that from 2010 to 2018, the number of discharges from hospitals, beds, health technicians and the total expenditure in Sichuan Province had an upward trend year by year. The total number of visits increased year by year from 2010 to 2013, dropped sharply from 2013 to 2014, then increased slowly from 2014 to 2018.

**Table 2.** Descriptive statistics of inputs and outputs

Year	Statistics	Total number of visits	Number of discharges	Number of beds	Number of health technicians	Total expenditure
2010	Mean	18 231 479.84	527 947.89	15 093.47	16 156.95	303 931.63
	SD	15 576 299.68	456 341.81	13 833.60	18 517.62	459 684.00
	Min	2 454 596.00	92 125.00	3026.00	3427.00	50 967.00
	Max	76 443 965.00	2 287 967.00	69 459.00	90 800.00	2 176 369.00
2011	Mean	19 776 541.32	556 369.84	16 761.11	17 664.11	384 497.74
	SD	17 686 374.55	517 397.90	15 970.04	20 679.05	591 621.73
	Min	2 397 163.00	95 579.00	3292.00	3634.00	63 569.00
	Max	86 772 382.00	2 568 559.00	79 780.00	101 030.00	2 796 424.00
2012	Mean	21 550 531.00	683 592.53	19 533.63	19 456.16	488 822.53
	SD	19 403 856.57	596 594.23	18 432.99	22 648.41	718 674.29
	Min	2 622 660.00	101 934.00	3700.00	3991.00	72 235.00
	Max	95 204 063.00	2 969 223.00	92 062.00	110 795.00	3 412 028.00
2013	Mean	31 593 879.53	715 074.11	21 313.63	21 307.95	576 138.95
	SD	46 201 934.85	624 172.98	20 241.73	24 529.26	849 693.48
	Min	2 610 717.00	114 082.00	3883.00	4710.00	97 853.00
	Max	202 075 265.00	3 116 138.00	100 957.00	120 091.00	4 034 817.00
2014	Mean	22 490 298.74	734 904.26	22 881.89	22 540.68	654 992.05
	SD	22 378 400.20	664 020.47	21 622.81	26 103.57	978 354.97
	Min	2 608 169.00	177 372.00	4272.00	5060.00	110 766.00
	Max	109 265 988.00	3 290 863.00	108 031.00	127 810.00	4 640 561.00
2015	Mean	22 781 246.74	763 033.53	24 325.84	23 513.47	757 096.37
	SD	23 201 891.72	700 094.23	22 965.47	27 658.17	1 167 092.01
	Min	2 548 596.00	112 285.00	4435.00	5518.00	133 340.00
	Max	113 379 760.00	3 485 771.00	114 726.00	135 131.00	5 520 047.00
2016	Mean	23 400 862.47	810 165.95	25 823.74	24 648.95	852 959.21
	SD	26 297 433.15	792 873.90	25 943.42	30 604.94	1 336 605.30
	Min	2 810 492.00	118 497.00	4447.00	6091.00	153 456.00
	Max	127 622 388.00	3 907 746.00	128 058.00	148 354.00	6 303 918.00
2017	Mean	24 416 791.84	891 771.37	28 000.11	26 371.42	966 968.47
	SD	27 979 782.41	848 164.64	27 210.91	32 605.47	1 493 132.22
	Min	2 797 648.00	120 371.00	4551.00	6316.00	163 892.00
	Max	135 645 090.00	4 192 201.00	134 507.00	158 002.00	7 047 097.00
2018	Mean	26 084 848.11	906 987.79	29 804.63	27 972.58	1 072 626.79
	SD	29 960 119.84	892 477.97	28 973.53	34 865.30	1 700 664.84
	Min	2 819 830.00	116 107.00	4899.00	6723.00	180 540.00
	Max	145 130 574.00	4 404 433.00	143 248.00	168 682.00	8 005 439.00

### A static analysis of the efficiency of regional health resources

Because the return to scale of resource allocation efficiency is generally variable,^26^ this paper uses the SE-SBM-VRS model to measure the health resource allocation of 19 cities in Sichuan Province from 2010 to 2018 (Table 3). The average super efficiency score of the sample is 1.041, which is DEA effective. Except for 2013 (0.683), the annual average super efficiency was >1, and the year with the highest average efficiency score was 2017 (1.165). The average regional super efficiency score is between 0.651 and 3.244. The cities that most need to improve the efficiency of resource allocation are Panzhihua (0.651), Guangyuan (0.663), Leshan (0.688) and Ya'an (0.698).

**Table 3.** SBM analysis of regional health resources in Sichuan Province from 2010 to 2018

Province	2010	2011	2012	2013	2014	2015	2016	2017	2018	Mean
Chengdu	2.930	3.271	3.177	1.614	3.329	3.527	3.707	3.729	3.916	3.244
Zigong	0.805	0.729	0.764	0.191	0.726	0.785	0.789	0.793	0.855	0.715
Panzhihua	0.553	0.551	0.583	0.120	0.633	0.707	0.713	1.002	1.001	0.651
Luzhou	1.018	1.012	1.073	1.034	1.033	0.897	0.871	0.867	1.023	0.981
Deyang	0.833	0.753	0.817	0.257	1.010	1.018	1.047	1.064	1.112	0.879
Mianyang	0.777	0.830	0.823	0.536	0.858	0.928	1.008	1.025	1.011	0.866
Guangyuan	0.797	0.715	0.735	0.145	0.418	0.768	0.765	0.803	0.821	0.663
Suining	0.785	0.889	0.887	1.825	0.847	0.916	0.854	0.874	0.900	0.975
Neijiang	1.012	0.771	0.705	0.230	0.707	0.741	0.794	0.846	0.822	0.736
Leshan	0.786	0.680	0.707	0.189	0.723	0.752	0.752	0.804	0.801	0.688
Nanchong	1.127	1.076	1.033	1.031	1.065	1.053	1.049	1.027	1.034	1.055
Meishan	1.056	1.076	1.027	1.018	1.037	1.026	0.751	0.841	1.018	0.983
Yibin	1.019	0.793	0.712	0.313	0.736	0.788	0.825	0.860	1.001	0.783
Guangan	0.876	0.803	1.015	1.054	0.895	1.013	1.030	1.028	0.861	0.953
Dazhou	0.852	0.852	0.759	1.010	0.781	1.006	1.048	1.059	1.033	0.933
Yaan	1.029	0.749	0.726	0.152	0.661	0.719	0.659	0.728	0.862	0.698
Bazhong	0.899	0.839	0.834	0.230	0.817	1.004	0.901	0.906	0.916	0.816
Ziyang	1.165	1.218	1.212	1.019	1.174	1.172	1.155	1.134	1.120	1.152
Aba	2.107	2.096	2.147	1.000	2.202	2.215	2.166	2.108	2.036	2.009
Mean	1.075	1.037	1.039	0.683	1.034	1.107	1.099	1.131	1.165	1.041

### Inter-period analysis of the efficiency of regional health resources

As shown in Table 4, the average TFP of the health resource allocation efficiency in Sichuan Province from 2010 to 2018 is 0.950, which is <1, indicating that the resource allocation efficiency in Sichuan Province is on a decreasing trend. Specific to individual years, only the total production factors of 2011–2012 and 2016–2017 are >1. The year with the highest TFP was 2011–2012 (1.003), and the year with the lowest TFP was 2013–2014 (0.878).

**Table 4.** Malmquist index analysis of regional health resource efficiency

	EFFCH	TECHCH	PECH	SECH	TFPCH
2010–2011	0.963	0.933	0.986	0.976	0.899
2011–2012	1.020	0.983	1.005	1.014	1.003
2012–2013	0.950	1.039	0.976	0.974	0.987
2013–2014	1.042	0.843	1.017	1.025	0.878
2014–2015	1.033	0.878	1.041	0.992	0.907
2015–2016	1.020	0.957	0.988	1.033	0.976
2016–2017	1.017	0.983	1.012	1.004	1.000
2017–2018	1.013	0.945	1.013	0.999	0.957
Mean	1.007	0.943	1.005	1.002	0.950

Abbreviation: EFFCH, technical efficiency change; TECHCH, technical progress index change; PECH, pure technical efficiency change; SECH, scale efficiency change; TFPCH, total factor productivity change.

**Figure 1.**
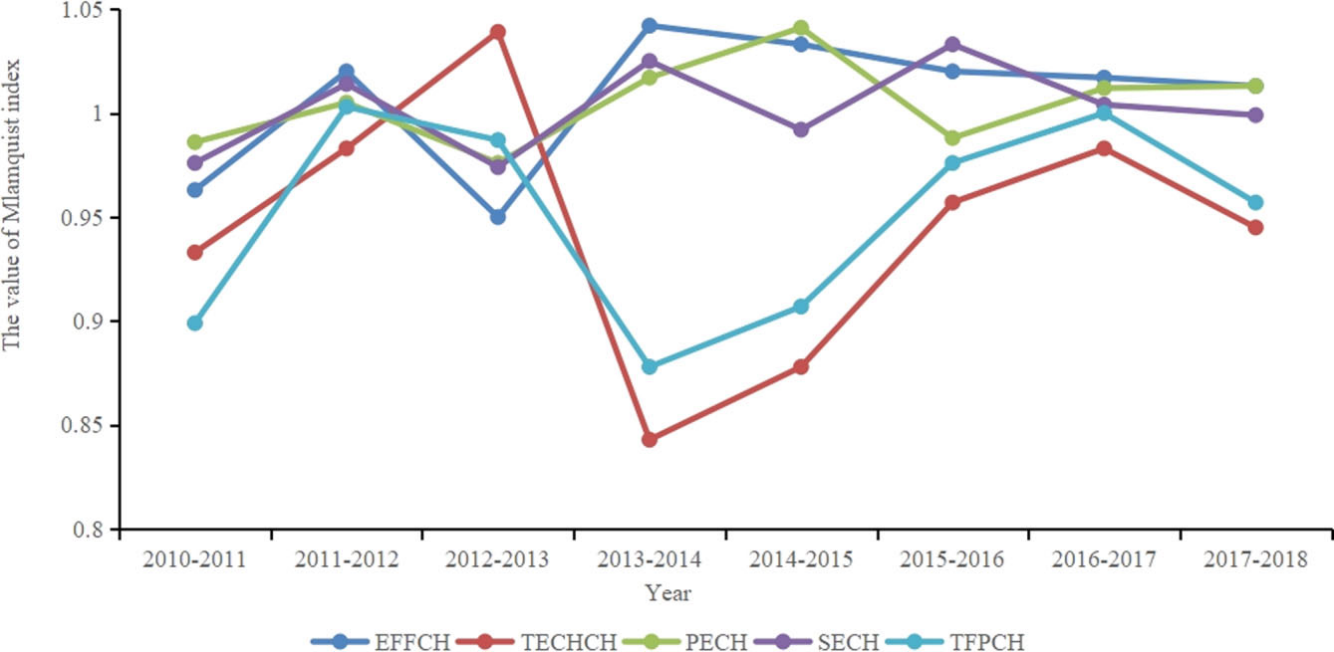
Interannual analysis of regional health resource efficiency.

**Figure 2.**
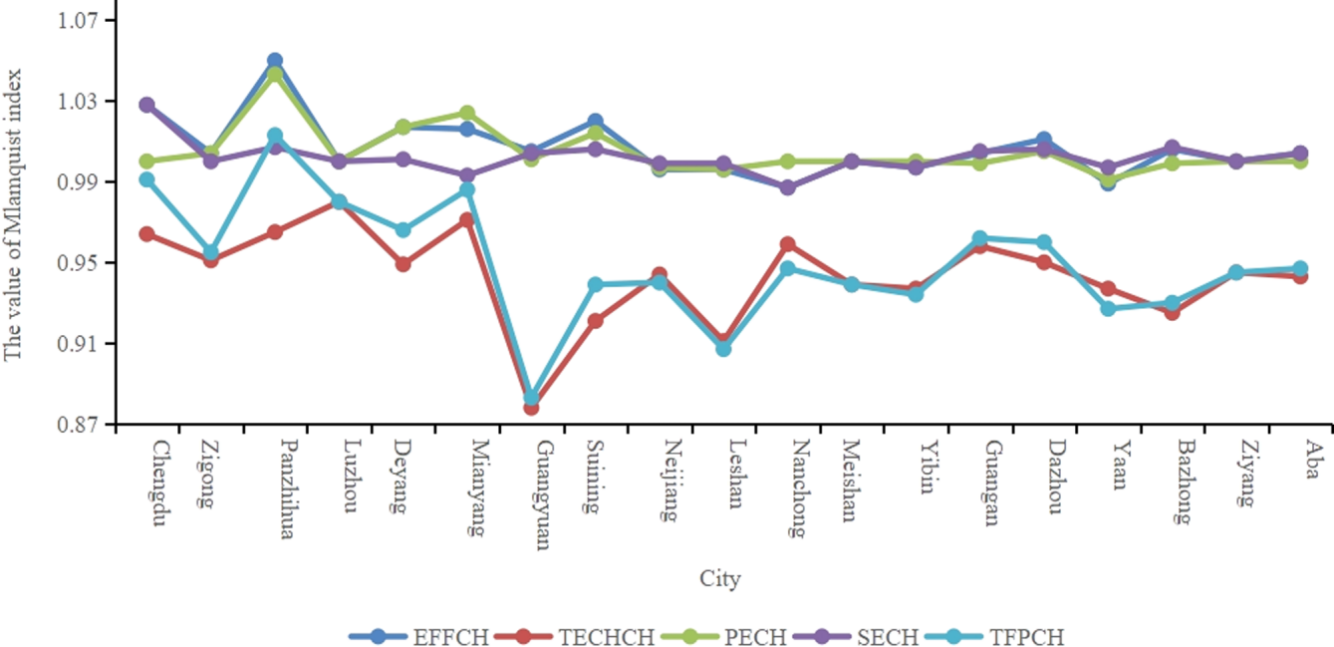
Analysis on the efficiency of regional health resources at the city level.

As shown in Figure 1, the TFP rate has two peaks and one trough. Two peaks occurred in 2011–2012 and 2016–2017, and one trough occurred in 2013–2014. Except for 2012–2013, the values of EFFCH in other years are higher than the value of TECHCH. Figure 2 shows the average value of the Malmquist index for 19 cities from 2010 to 2018. Only Panzhihua has a TFP>1 and the city with the lowest total factor production rate is Guangyuan (0.883).

### The input-output optimization plan for the allocation of regional health resources in 2018

Table 5 shows the analysis of input redundancy and output shortage in 2018. Based on the super efficiency score, the city with a score of <1 is the invalid resource allocation area. The input-output optimization scheme of health resource allocation in Sichuan Province in 2018 is analyzed by the SE-SBM model. In 2018, the resource allocation efficiency score of eight cities was <1, among which the lowest score was Guang'an (0.80). In the input index, the 2.63% bed number and 4.11% total expenditure should be reduced to increase by 44.21% the total number of visits. What is more, the highest proportion of input and output indicators are total expenditure and the total number of visits.

**Table 5.** Analysis of input redundancy and output shortage in 2018

	Inputs			Outputs	
	Number of beds	Number of health technicians	Total expenditure	Total number of visits	Number of discharges
Zigong	200.61(0.91%)	267.74 (1.37%)	59 708.62 (8.67%)	5 162 459.05 (25.51%)	0.00
Guangyuan	797.13 (3.61%)	0.00	27 038.30 (4.34%)	6 414 791.44 (37.05%)	0.00
Suining	461.68 (2.29%)	1731.58 (9.99%)	75 890.59 (12.71%)	767 027.01 (3.82%)	0.00
Neijiang	357.36 (1.48%)	720.09 (3.64%)	0.00	6 049 544.14 (39.10%)	0.00
Leshan	633.72 (2.63%)	0.00	28 544.39 (4.11%)	7 759 109.26 (44.21%)	0.00
Guangan	0.00	159.35 (1.01%)	17 264.82 (3.20%)	4 652 788.33 (28.95%)	0.00
Yaan	0.00	1153.83 (9.66%)	24 016.10 (6.55%)	1 222 902.17 (11.80%)	26 739.70 (7.80%)
Bazhong	1689.54 (7.76%)	1185.63 (7.06%)	0.00	1 425 025.98 (7.60%)	0.00

### Tobit analysis of health resource allocation

Using the Tobit regression model, the current study analyzed the influencing factors of health resource allocation efficiency in Sichuan Province from 2010 to 2018. The results are shown in Table 6. The average annual income of residents (p=0.001), population density (p<0.001) and educational level (p<0.001) have positive significance for the allocation efficiency of health resources, and the per capita GDP and urbanization rate have no significant effect on the efficiency.

**Table 6.** Tobit regression analysis of the allocation efficiency of health resources (N=171)

Variable	coefficient	SE	T-ratio	p	95% CI
PGDP	0.048	0.101	0.47	0.637	(−0.152 to 0.247)
The average annual income of residents	0.169	0.052	3.24	0.001	(0.066 to 0.271)
Urbanization rate	−0.022	0.100	−0.22	0.828	(−0.219 to 0.176)
Population density	0.246	0.049	5.04	<0.001	(0.149 to 0.342)
Education	0.153	0.043	3.56	<0.001	(0.068 to 0.238)
constant	1.041	0.039	26.67	<0.001	(0.964 to 1.118)
Sigma	0.261	0.028			(0.210 to 0.323)
Log-likelihood	−127.654				
X^2^	78.95				
Prob>X^2^	<0.001				

Abbreviation: PGDP, per capita gross domestic product.

## Discussion

To address the difficulty of access to healthcare, China carried out a deep reform of the health system in 2009. This study calculates the efficient allocation of health resources in Sichuan Province from 2010 to 2018, which can explain to some extent the effect of the health system reform that started in 2009 and can also provide partial suggestions for future health reforms. In addition, Sichuan Province is located in the southwest, has the second largest hospital in the country in terms of comprehensive strength and absorbs a large number of patients from the southwest, so it is representative to choose Sichuan Province for analysis.

As a whole, the allocation of health resources in Sichuan Province is increasing every year because of the increasing population and the increasing demand for health services. In response to the WHO's demand for everyone to enjoy essential health services, China has carried out many comprehensive medical reforms since 2009, including increasing the number of health resources to improve people's access to health services.

Using the super efficiency SBM for the calculation, it was found that the sample's average super efficiency score is 1.041, indicating that the resource allocation efficiency in Sichuan Province is relatively good. However, the average super efficiency value (3.244, 0.651) between cities has a large gap and regional differences. Moreover, resource allocation efficiency in 2018 has improved compared with 2010, and the proportion of cities with effective resource allocation has increased from 45% to 60%. The average annual excess efficiency was >1 except for in 2013, which may be due to two comprehensive medical reforms in Sichuan Province in 2009 and 2016. The health resource allocation efficiency in 2013 scored the lowest among all years, perhaps because of the magnitude seven earthquake in Ya'an City, Sichuan Province, in April 2013, and the heavy rainstorm in Sichuan Province in July. Natural disasters indirectly lead to the inefficiency of resource allocation. As a result, we are supposed to do an excellent job of early warning prevention and control mechanisms to reduce its adverse effects. Besides, the super efficiency value of Aba is >2, indicating that perhaps because of its vast area and relatively scattered population, the government ensured that residents have access to better medical services by vigorously developing primary medical and health institutions.

According to the Malmquist index, the average TFP of health resource allocation efficiency in 2010–2018 was <1, indicating that the resource allocation efficiency of Sichuan Province is on a decreasing trend. TFP can be decomposed into the technical efficiency index and technological progress index, while the technical efficiency index can be decomposed into pure technical efficiency and scale efficiency. It can be seen from Figure 1 that the improvement of overall efficiency TFP in this study stems from the improvement of the technical efficiency index EFFCH because the TECHCH of the technological progress index was only >1 in 2012–2013, which shows that the level of organization and management of resource allocation in Sichuan Province is better. It is urgent to improve technological progress and innovation in the health industry.^27^ Some scholars have pointed out that technological innovation capabilities may be related to health technicians' educational level and the use of health facilities.^21,28^ Moreover, as shown in Table 4, the improvement of technical efficiency EFFCH is mainly due to the improvement of the pure technical efficiency change (PECH). The scale efficiency change (SECH) is the leading cause of lower technical efficiency, so Sichuan Province needs to improve its scale efficiency and allocate resources scientifically.

Based on the relaxation of SBM and the analysis of eight cities with ineffective resource allocation efficiency in 2018, it can be seen that the redundancy of total expenditure of Sichuan Province is the highest, followed by the redundancy of health technicians. Taking Suining City as an example, the total expenditure redundancy is as high as 12.71%, and the redundancy ratio of the number of health personnel is 9.99%. Therefore, these cities need to make effective use of health expenditure and improve the technical level of health technicians to increase the number of visits to health institutions.

Regarding the influencing factors on the efficiency of health resource allocation, some studies have shown that per capita GDP has a positive effect on the production efficiency of health resources,^5,29,30^ while this study shows that per capita GDP and the urbanization rate have no significant effect on efficiency, which may be due to government policy weakening the impact in this area.^19,28^ In addition, Tobit results show that the average annual income of residents, population density and education level have a substantial impact on the efficiency of health resource allocation, which is consistent with the research results of Guo et al. and others.^31,32^

## Conclusion

This study used the super efficiency SBM model and the Tobit model to analyze the efficiency of health resource allocation and its influencing factors in 20 cities in Sichuan Province from 2010 to 2018 and reached the following conclusions: first, the total amount of health resource allocation in Sichuan Province has gradually increased since 2010. The average health resource allocation efficiency in 2018 showed a slow upward trend, but there are regional differences. Second, the Malmquist index shows a downward trend in TFP from 2010 to 2018, mainly because of insufficient technological innovation capacity and unreasonable resources allocation. Third, the allocation efficiency of health resources is affected by the average annual income of residents, population density and education level. To sum up, to improve resource allocation efficiency, we need to focus on the key factors of natural disasters, economic factors, population density and education level.

## Supplementary Material

ihac054_Supplemental_File

## Data Availability

The data were extracted from the 2010–2018 *Statistical Yearbook of Sichuan Province* and *Health Yearbook of Sichuan Province*.
