# Supplementary material for: The efficiency of health resource allocation and its influencing factors: evidence from the super efficiency slack based model-Tobit model
Source: Int Health. 2022 Aug 13;15(3):326–34. doi: 10.1093/inthealth/ihac054 (PMC10153566; doi:10.1093/inthealth/ihac054)
Supplement: ihac054_Supplemental_File [file ihac054_supplemental_file.docx]

**Supplementary Material**

**Abbreviations**

| DEA | Data Envelopment Analysis |
| --- | --- |
| SE-SBM | Super efficiency slack based model |
| DMU | Decision-making unit |
| CCR | Charnes, Cooper, and Rhodes |
| BCC | Banker, Charnes, and Cooper |
| CRS | constant returns to scale |
| VRS | variable return to scale |
| MI | the Malmquist Index |
| TFP | total factor productivity change |
| TECHCH | technical progress index |
| EFFCH | technical efficiency change |
| PECH | pure technical efficiency change |
| SECH | scale efficiency change |
| GDP | Gross d population |
